# Relationship between a three-month physical conditioning “posture-balance-motricity and health education” (PBM-HE) program on postural and balance capacities of sedentary older adults: influence of initial motor profile

**DOI:** 10.1186/s11556-018-0203-0

**Published:** 2018-11-20

**Authors:** Pierre Louis Bernard, Hubert Blain, Aurelie Gerazime, Olivier Maurelli, Jean Bousquet, Grégory Ninot

**Affiliations:** 1 Euromov, University of Montpellier, 700 avenue du Pic Saint Loup, Montpellier, France; 2 Department of Internal Medicine and Geriatrics, Antonin Balmes Center, University Hospital of Montpellier, Montpellier, France; 3 EA 4556 Epsylon, University of Montpellier, 4 boulevard Henri IV, Montpellier, France; 4 MACVIA-LR. European Innovation Partnership on Active and Healthy Aging Reference Site, 34000 Montpellier, France

**Keywords:** Balance, Older adults, Frail, Training program

## Abstract

**Background:**

The aims of this study were (i) to define the relationship between a physical reconditioning cycle using balance exercises and muscular-articular stress and the balance capabilities of sedentary older adults and (ii) to assess whether older adults with weaker equilibrium abilities have a significantly limited progression. Our sample consisted of 338 people (263 women, 75 men) with an age, weight and height of 74.4 years (+/− 8.6), 67 kg (+/− 13.6) and 161.4 cm (+/− 8) and with a body mass index of 25.6 (+/− 4.3). The functional evaluations consisted of individual motor profile tests, monopodal eyes open and eyes closed for 30 s, a Timed Up and Go test (TUG) and stabilometric measurements on hard ground with eyes open for a duration of 25.6 s. The physical repackaging protocol was based on the 12-week Posture-Balance-Motricity and Health Education (PBM-ES) method with two 90-min weekly group sessions.

**Results:**

The evolution of the “posture” and “balance” variables was significantly associated with the equilibration capacities (*p* < 0.001). For unipedal stance with open eyes on the dominant and non-dominant sides, respectively, the progressions were significant for the profiles of middle (OR: 4.78 and 2.42) and low levels (OR: 4.34 and 1.66). Eyes-closed progressions were non-significant for the low-level balance profiles. For the COP Surface and Length variables, compared to those with high levels of balance, respectively, the progressions were significant for the middle- (OR: 1.41 and 2.98) and low-level (OR: 2.91 and 3.28) profiles.

**Conclusions:**

After a 3-month bi-weekly PBM-HE program, we observed that sedentary older adults with the lowest initial level of balance progressed significantly more than high-level individuals, but only for basic motor abilities. It turns out that even among the most deconditioned people and older adults, very significant progress can be made. This increase requires an individualized training content focused on initial mobilizable capacities.

## Background

The set of functions on which locomotion and balance depend is influenced by aging. The mechanisms of aging impact both the cognitive and sensory-motor spheres [1–3] and disrupt the ability to adapt. The integrity of the visual-vestibular and muscular-articular structures responsible for the perception stage decreases with age [4]. In addition, cognitive decline and lowering of executive functions contribute to the loss of efficiency of postural and locomotor functions and of skills in daily life activities [5, 6].

In order to limit the effects of aging and optimize the functional abilities of the elderly, the use of physical activities is often mentioned [7–9]. Critical analyses were conducted to define the respective effects of physical activity programs on the balancing abilities of seniors. The majority of reference programs to improve balance include static and dynamic motor exercises with regulation of difficulty levels in order to optimize adaptive capabilities [10, 11].

To achieve this goal and to increase posture and balance capacities, some programs used a specific physical activity such as brisk walking [12, 13], balance training [14, 15], serious gaming with or without force-plate [16, 17], muscular conditioning [18, 19], tai-chi or pilates [20]. Other programs combined different motor activities [21–24].

Beyond the optimization of balance capacities, the prevention of autonomy loss through physical activity programs is now one of the main objectives for older adults, taking medical, social and economic consequences into account. Some studies have shown that different programs are more effective in preventing and reducing falls [25–27]. In addition, the practice environment, such as a community or medical care organization, often characterized by individuals older in age and suffering from frailty, may justify the difficulty of reaching the fall prevention target. The potential optimization of functional capabilities in frail people is now considered a major issue and constitutes the overall objective of this study.

It is considered that the improvement of state of health requires a multifactorial approach. In non-medicated interventions, the combination of physical activity and health education would be a preferred approach in maintaining and improving health. The combination of motor activities and health education with active lifestyle and quality nutrition leads to healthy behaviors [28, 29]. We favored this approach within the PBM-HE method by associating 30 min of HE with 60 min of physical activity within each session [30, 31].

In addition to the analysis of the potential improvement of the balancing capacities, whatever the age, another concern consists of the functional reserves available to the person and which partially justify measured progress. This present question in the field of learning and cognitive science also applies to the aging sector and refers to notions of adaptation and frailty [32, 33]. Some studies describe the fragility of the elderly person in various physiological, psychological or neuro-cognitive domains and identify markers such as walking to determine the health and future of the person [34–37]. However, in the motricity domain, few studies quantify the influence of the initial level, which can be considered as a significant lowering of functional reserves on the potential recovery capacities and the measured benefits.

The purpose of this study was to observe the relationship between:

1. a physical reconditioning program using static and dynamic balance solicitations, some muscular-articular exercises and information on health education and the balancing capacities in older adults,
2. a physical reconditioning program and the ability of people to progress with weaker balance capacities.

## Method and procedure

This study was carried out within the “Preservation of the Autonomy of Older Adults” program of the Regional Health Organization of Languedoc-Roussillon. We analyzed the effect of the three-month PBM-HE training program on sedentary older adults. Initial and terminal functional evaluations were performed in geriatric and gerontological settings engaged in this study. The study was validated by the Internal Review Board n° 1711B of the Euromov Research Center (University of Montpellier).

### Design

We performed a prospective investigation study without a control group. The participants were not randomized. The data were collected before and after the program. The participants were assigned to three graded groups related to functional capacities.

### Participants

The participants were recruited during a public meeting dedicated to fall prevention. They were invited on the basis of health, age and place of residence criteria by the public health services in the region. Following the public meeting, they were invited to participate in a functional capacity assessment session to meet the inclusion criteria. People were eligible if they met the following criteria: (1) not diagnosed with osteoarthritis, rheumatoid arthritis, ischemic heart disease, previous joint replacement surgery, cerebrovascular disease affecting lower limb function, (2) without pain and not taking medication known to alter physical performance. A medical visit checked the criteria for non-inclusion and validated a certificate of access to the practice cycle.

Our population consisted of 338 people, 263 women (78%) and 75 men (22%). They had an average age of 74.4 years (+/− 8.6), an average weight of 67 kg (+/− 13.6) and an average height of 161.4 cm (+/− 8). The average body mass index was 25.6 (+/− 4.3). The inclusion criteria were: (i) age greater than 60 years, (ii) unipedal stance of less than 5 s, (iii) complaint in balance, (iv) weekly physical activity of less than two hours and (v) a medical prescription of non-indication to physical activity. The exclusion criteria were motor impairment of the lower limbs and neurocognitive impairment that disrupted understanding and exercise. Participation was voluntary, with a newsletter and informed consent and without financial reward.

### Outcome measures

#### “Posture-balance-motricity” evaluation

The dimensions of posture, balance and motricity were evaluated by ten specific motor exercises. Each dimension was tested on 30 points and the addition of the three dimensions constituted the “individual motor profile” (IMP), tested on 90 points [30, 31].

#### Unipedal stance

The unipedal stance was performed for 5 s in four conditions with the eyes open on the dominant and non-dominant sides. It was then performed under the same conditions with eyes closed. Thirty seconds of recovery were applied between each condition. Each person was placed standing in front of the table and behind the investigator to prevent falling. The upper limbs were free of movement and the lower limb was suspended [38].

#### Timed up and go test (TUG)

This dynamic balance test consisted of participants standing up from a seated position and walking for 3 m until they reached a landmark. Once the landmark was reached, they were to turn around it and return back to the initial starting point and seated position [39].

#### Stabilometric evaluation

The environment satisfied the norms for postural evaluation regarding dimensions, brightness and environmental noise. The experimental situation respected the norms for static postural evaluation on a hard floor [40]. Participants had to maintain quiet stance conditions while standing on a 530x460x35-mm force platform (Medicapteurs SFP® “40 Hz/16b”) equipped with three pressure sensors (hysteresis < 0.2%). Signal processing was accomplished with a 16-bit A/D converter at 40 Hz. Feet were oriented at the angle of 15° from the sagittal midline, with heels 4 cm apart. Arms were held alongside the body and participants were asked to focus on a visual reference mark fixed 100 cm in front of them. The recordings started 5 s after the beginning of each test, and lasted for 25.6 s.

Participants were assessed in eyes-open (EO) conditions. The main dependent variables were the surface (Surf) and the length (Lg) of the stabilogram, the mean along the x-axis (Xm) and the mean along the y-axis (Ym). Surf was taken to represent the area of the ellipse that best fitted the COP displacement at the 95% confidence interval. Lg represented the total length of the COP path, defined as the sum of the distances between all consecutive points on the COP path.

### Adapted physical activity program (APAP)

The APAP was based on the “Posture-Balance-Motricity and Health Education” (PBM-HE) program [30, 41, 42]. This program consisted of motor activities and health information focusing on the health and quality of life goals for people with a loss of autonomy. The PBM-HE training consisted of two 90-min weekly sessions for three months. The sessions were divided into 30 min of health education and 60 min of motor exercises. The first two sessions and the last two sessions focused on evaluation. Each session was composed of 10 min of warm-up, 40 min centered on functional capabilities (IMP) and 10 min of recovery.

The exercises focused on the sensorimotor dimensions of balance centered on cutaneous and proprioceptive factors and on the muscular-articular factors engaged in perception as well as on motor adaptations. For planning sessions and exercises, PBM follows the main dimensions of:

- Physical: static/dynamic, single-articular/multi-articular, concentric/eccentric, unipedal/bipedal,
- Sensorial: hard/soft ground, rough/smooth ground, open/closed eyes, shod/barefoot, flat/varied floor, forwards/backwards,
- Cognitive: with/without temporal constraints, with/without spatial constraints, single multiple tasks,
- Relational: individual/group exercises, collaboration/opposition exercises,
- Physiological: with/without cardiac constraint (heart rate greater than 20/30 beats/min to resting heart rate value), with/without ventilatory constraint,
- Psychological: with/without uncertainty, with/without encouragement, with/without constraint (temporal/spatial).

The Health Education (HE) part of the PBM-HE program is based on the social-cognitive behavior change theory applied in the heath sector [43]. The main message is individual health appropriation. It was carried out in an appropriate balance between the health promotion behaviors already applied by some of the people in the group and the information provided by scientific societies [44], scientific collective assessments and health authorities [45, 46]. The main topics related to health and the prevention of falls were (1) nutrition, (2) health practices (3) drugs (4) habitat and environment adaptations (5) physical and psychological resources against sedentariness. Twenty-four sessions were organized in the form of a workshop and only one message was delivered at each session. During the workshop, people individually reformulated and contextualized the information in everyday life.

### Statistical analysis

First, we analyzed the influence of the training program on the functional capacities of the 338 participants. The normality of the distribution was tested by the Kolmogorov-Smirnov test. A *t*-test for paired series was used to compare the values of initial and terminal functional evaluations. The level of statistical significance was set at *p* < 0.05. The analyses were performed with Statview 5.0. Then, we tested the hypothesis that older adults with lower balance are characterized by a significant limited evolution. The balance parameter was created with the data obtained by the initial measurement in the TUG test. Those with good balance (considered as the baseline) had a TUG of less than 14 s and accounted for 74% of the total sample. Twelve percent had an average TUG test of between 14 and 20 s, while the remaining 14% had a bad balance status with a TUG of more than 20 s.

Some explanatory variables were also created with the differences between initial and terminal evaluations for the PBM, unipedal stance and stabilometric tests. A polytomous logistic regression was used to estimate the odd ratio (OR) and a 95% CI. A qualitative response variable using 3 modalities (high, middle and low levels of balance) was created to evaluate the means of other variables. A bootstrapping method was used to overcome the limits of the *p*-value and the confidence interval was significantly zero. This is a resampling method for making a statistical inference by drawing lots with repeat delivery [47, 48]. This made it possible to increase the sample size, and to refine the meaning of the analysis. Univariate and multivariate analyses were performed using the Cox’s proportional hazards model. Results from the Cox regression were internally validated using the bootstrap procedure, generating a total of 100 replicates. We used a method of internal validation of the results obtained following the polynomial logistic regression. Initial proportions have been preserved [49, 50]. The analysis was performed with SAS 9.1 software.

## Results

### Influence of the training program

**Fig. 1:**
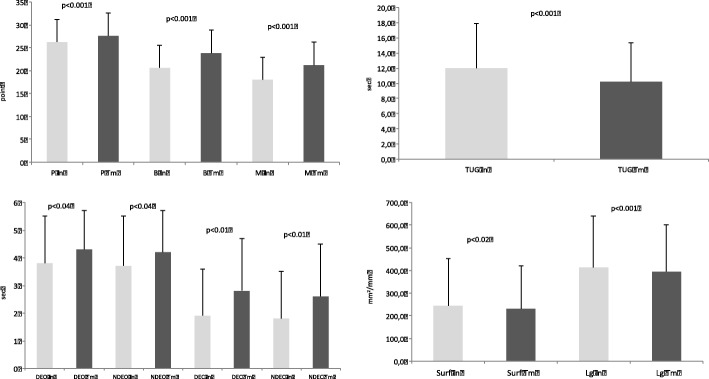
functional capacities measured during initial (In) and terminal (Tm) evaluations. P: Posture, B: Balance, M: Motricity tests. TUG: Timed Up and Go test. DEO: dominant side eyes opened, NDEO: non-dominant side eyes opened, DEC: dominant side eyes closed, NDEC: non-dominant side eyes closed. Surf: surface of the center of pressure, Lg: length of the center of pressure

We observed a significant positive evolution of all parameters measured (Fig. 1). The three dimensions of “Posture-Balance-Motricity” increased significantly (*p* < 0.001) and the time taken in the TUG test decreased significantly (*p* < 0.001). Unipedal time analysis showed significant evolution in both of the eyes-open conditions (*p* < 0.004) and both of the eyes-closed conditions (*p* < 0.001). For stabiometric evaluation with eyes open, we observed a significant decrease of the surface (*p* < 0.02) and the length (*p* < 0.001) of the COP.

### Influence of the initial level

#### PBM evaluation

The progression of the “posture” parameter was significantly associated with the sedentary older adult’s balancing abilities (*p* < 0.001). Compared to people with a high level of balance, people with low and middle levels showed an increase in this parameter (OR: 11.65 and 8.83 respectively). For the “balance” parameter, progression was also significantly associated to balance capacities (*p* < 0.001). People with a middle level of balance showed a significant increase compared to the high-level group (OR: 2.49), whereas we did not observe any increase for people with a low level of balance. For the “motricity” parameter, for the low- and middle-balance subgroups, we did not observe a significant difference with the high-level group. The decrease of the “motricity” parameter (Mtm < Min) affected 10% of the people with a middle level of balance and was a minor trend (OR: 1.95, IC: 1.68–2.28). For the IMP, we observed a significant increase only for the high-level balance group (OR: 1.49, CI95%: 1.28–1.74) (Table 1).

**Table 1:** Evolution, odd ratio (OR) and significance for the Posture (P), Balance (B), Motricity (M) and Individual Motor Profil (IMP) of PBM method for the low, middle and high levels of balance groups

		High balance (*n* = 250)	Middle balance (*n* = 40)		Low balance (*n* = 48)		*P*-value
		%	%	OR (IC 95%)	%	OR (IC 95%)	
Posture	Ptm < Pin	7	3	1.55 (1.25–1.91)		–	< 0.001
	Ptm = Pin	55	14		11		
	Ptm > Pin	38	83	8.83 (8.05–9.70)	39	11.65 (10.55–12.86)	
Balance	Etm < Ein	9	7	1.91 (1.59–2.30)	6	0.60 (0.52–0.70)	< 0.001
	Etm = Ein	11	5		14		
	Etm > Ein	80	88	2.49 (2.15–2.88)	80	0.82 (0.74–0.89)	
Motricity	Mtm < Min	6	10	1.95 (1.68–2.28)	13	1.03 (0.92–1.17)	< 0.001
	Mtm = Min	11	9		23		
	Mtm > Min	83	81	1.11 (0.99–1.25)	64	0.34 (0.32–0.38)	
IMP	PMItm < PMIin	7	5		7		< 0.001
	PMItm > PMIin	93	95	1.49 (1.28–1.74)	93	0.94 (0.83–1.07)	

*in* Initial evaluation, *tm* Terminal evaluation

#### Unipedal stance

For the four experimental conditions of the unipedal stance test, evolution was significantly associated with balance capacities (*p* < 0.001) (Table 2). For the eyes open on the dominant side (EOD) test, 51% of those with a middle level of balance increased their stance (OR: 4.78), as did 49% of those with a low level of balance (OR: 4.34). For the eyes open on the non-dominant side (EOND) test, 43% of those with a middle level of balance increased their stance (OR: 2.42), as did 30% of those with a low level of balance (OR: 1.66).

**Table 2:** Evolution, odd ratio (OR) and significance for unipedal stance test on dominant side (D) and non-dominant side (ND), eyes opened (EO) and eyes closed (EC) for the low, middle and high levels of balance groups

		High balance (*n* = 250)	Middle balance (*n* = 40)		Low balance (*n* = 48)		*P*-value
		%	%	OR (IC 95%)	%	OR (IC 95%)	
DEO	DEOtm < DEOin	4	12	5.44 (4.83–6.13)	11	4.79 (4.25–5.39)	< 0.001
	DEOtm = DEOin	75	37		40		
	DEOtm > DEOin	21	51	4.78 (4.44–5.14)	49	4.34 (1.04–4.65)	
NDEO	NDEOtm < NDEOin	4	7	2.91 (2.53–3.34)	21	8.54 (7.72–9.46)	< 0.001
	NDEOtm = NDEOin	71	50		49		
	NDEOtm > NDEOin	25	43	2.42 (2.26–2.59)	30	1.66 (1.54–1.79)	
DEC	DECtm < DECin	14	4	5.24 (4.48–6.12)	9	1.83 (1.52–2.19)	< 0.001
	DECtm = DECin	33	55		63		
	DECtm > DECin	53	41	2.13 (1.98–2.29)	28	0.60 (0.55–0.66)	
NDEC	NDECtm < NDECin	11	14	0.81 (0.73–0.90)	14	0.60 (0.54–0.66)	< 0.001
	NDECtm = NDECin	33	51		67		
	NDECtm > NDECin	56	35	0.39 (0.36–0.43)	19	0.16 (0.15–0.18)	

*in* Initial evaluation, *tm* Terminal evaluation

For the eyes closed on the dominant side (ECD) test, 53% of those with a high level of balance improved after training. Forty-one percent of people with a middle level of balance improved (OR: 2.13), whereas no significant increase was observed for the low level of balance group (OR: 0.60, IC: 0.55–0.66). For the eyes closed on the non-dominant side (ECND) test, 56% of people with a high level of balance improved. The other two balance profiles showed no significant evolution in the unipedal stance.

#### Kinematic stabilometric parameters

For the COP Surface, 58% of the people with a middle level of balance (OR: 1.41) and 64% of those with a low level of balance (OR: 2.91) improved compared to people with a high level of balance (Table 3). For the COP Length, we observed an improvement in 67% of the people with a middle level (OR: 2.98) and in 60% of people with a low level of balance (OR: 3.28).

**Table 3:** Evolution, odd ratio (OR) and significance for center of pressure parameters of Surface (Surf), Length (Lg), mean medio-lateral (X) and antero-posterior (Y) projections of COP during eyes opened test for the low, middle and high levels of balance groups

		High balance (*n* = 250)	Middle balance (*n* = 40)		Low balance (*n* = 48)		*P*-value
		%	%	OR (IC 95%)	%	OR (IC 95%)	
Surface	Surftm < Surfin	51	58	1.41 (1.27–1.56)	64	2.91 (2.57–3.30)	< 0.001
	Surftm = Surfin	16	13		7		
	Surftm > Surfin	33	29	1.08 (0.97–1.21)	30	1.95 (1.71–2.23)	
Length	Lgtm < Lgin	43	67	2.98 (2.67–3.34)	60	3.28 (2.91–3.69)	< 0.001
	Lgtm = Lgin	19	10		8		
	Lgtm > Lgin	38	23	1.18 (1.04–1.33) 32	32	1.99 (1.76–2.26)	
medio-lateral projection	Xtm < Xin	41	40	1.66 (1.49–1.85)	47	1.73 (1.57–1.91)	< 0.001
	Xtm = Xin	20	12		13		
	Xtm > Xin	39	49	2.10 (1.89–2.34)	40	1.54 (1.39–1.71)	
antero-posterior projection	Ytm < Yin	47	52	0.94 (0.84–1.04)	51	1.11 (0.99–1.23)	< 0.001
	Ytm = Yin	11	13		11		
	Ytm > Yin	42	35	0.71(0.63–0.79)	38	0.93 (0.83–1.03)	

*in* Initial evaluation, *tm* Terminal evaluation

For the projection of COP in the medio-lateral plan, for the middle level of balance group, 40% improved but 49% declined. This trend was confirmed also for people with a low level of balance. In addition, the training program did not favorize optimizing the postural regulation of COP in the antero-posterior plane in people with low and middle levels of balance.

## Discussion

The study investigated two assumptions of aging effects and the potential optimization of functional capacities following an adapted physical activity program. In a chronological approach, age could be considered as a limiting factor of the balance function, contrary to a biological approach that envisions adaptable functions whatever the age. The functional profile analysis, after three months of adapted physical activities, showed the benefits of this type of kinesthetic, postural and muscular-articular solicitation program on balance capabilities. These data confirm studies that demonstrated positive sensitive-motor adaptations in older adults after a training program [21, 51–53]. As far as postural and equilibration functions are concerned, we have compared our results to certain populations, to close training protocols and to similar measured parameters. Cadore et al. [54] analyzed the effect of multicomponent exercises for the muscle capacity of a group of institutionalized people aged 91.9 (+/− 4.1) composed of 70% females. They participated in 12 weeks of re-training, twice a week, with 40-min exercises focusing on leg extension and upper limb exercises. Exercise training significantly improved the time spent on the TUG. These data were confirmed by Kim et al. [55] for 131 women aged 80.7 (+/− 2.8) living in a community dwelling and following a physical comprehensive training program of 12 weeks with 2 sessions of 60 min per week. These data are also confirmed by other studies [15, 16, 19]. El-Khoury et al. (2015) reported significant effects of a 12- and 24-month physical activity program for 706 people. In their randomized-controlled trial study, they observed a significant improvement of TUG after 12 months (*p* = 0.03) and 24 months (*p* = 0.02). For the time in single leg stance, they also observed a significant improvement after 12 months (*p* = 0.006) and after 24 months (*p* < 0.001). These recent studies confirmed the numerous other studies about the ability to progress on these two postural and dynamic balancing capacities. Our results corroborated data and do not provide original information. However, the use of health education questions the specific place of this issue in the progress observed. Within a future RCT, a specific group benefiting only from health education could measure its impact. In addition, various studies quantified the effects of physical training on postural control tested by stabilometric method [8, 14]. In our study, we highlighted a significant improvement of surface and length of the COP. These data were regularly observed following various protocols [15, 56, 57], although authors have shown the limits of the COP to quantify progress [58, 59]. On the basis of the COP, the use of other treatment methods and other parameters could provide additional information [60, 61]. In addition, these data also confirmed the validity of the PBM-HE program to optimize the balance capacities of sedentary older adults who are at risk of falling [41, 42].

Beyond this longitudinal analysis of the total population, the main originality of this study was that people with lower balance capacities would have significantly limited progression compared to people with higher balance capacities. This assumption is partially invalid. Indeed, the analysis of the influence of the training program has shown a significant increase in balance capacities in older adults with a low level of balance compared to people with a high level of balance, but exclusively on motor basic behaviors and basic balance capacities. Beyond the evolution of each parameter significantly associated with the balance capacities of older adults (*p* < 0.001), we must consider specific adaptations. In fact, the PBM analysis showed an improvement for people with a low level of balance only on low complexity motor tasks. Thus, on the PBM scale, the “postural” dimension, which corresponds to static postural regulation, without plantar and visual disturbances, and without double cognitive tasks or significant muscular solicitations, shows a significant improvement for people with a low initial level of balance (OR: 11.65) and a middle initial level of balance (OR: 8.83). The progression of the “balance” dimension, which corresponds to a more complex dynamic regulation of balance, was only observed for the group with a middle level of balance (OR: 2.49) while the “motricity” dimension did not improve for low initial and middle levels of balance. This information was confirmed by stabilometric analysis performed with eyes open on a hard ground for 25.6 s. Compared to people with a high level of balance, 64 and 60% of people with a low level of balance improved respectively on the surface and length of COP parameters (OR of 2.91 and 3.28). To a lesser extent, these improvements were observed for people with a middle level of balance on surface (OR 1.41) and length (OR 2.98) of COP. This confirms the ability of the weakest to progress if the task is simple.

The results of the unipedal stance tests provide some additional details by confirming all the capabilities of sedentary older adults to improve their motor responses, but only within the complexity of the task to be performed. Indeed, considering more and more constraining of the four unipedal tests carried out with eyes open and closed on the dominant and non-dominant sides, we observed improvements only under eyes-open conditions. Only people with a middle level of balance improved during the unipedal test with eyes closed on the dominant side (OR 2.13) while neither of the two groups of low level and middle level of balance progressed with in the eyes closed on the non-dominant side. This progression of unipedal time with eyes closed was examined within the group with a high initial level of balance: on the dominant side, it was at 53% and on the non-dominnat side, 56%.

In a public health approach [62], the optimization of functional capacities, and more specifically of balance, in sedentary older adults was expected. In addition to the many studies demonstrating the benefits of adapted physical training programs on balance capacities of older adults [8, 14, 22], our results show that an initial level of motor skills does not systematically limit the improvement of physical health. These results confirmed two methodological considerations in the field of adapted physical activity. The first consideration concerns the metrological quality and the validity of the functional assessments of identification of the motor skills to be mobilized during training programs. The second consideration concerns the methodology of intervention with older populations. To date, in the gerontological approach where each person is unique, there are no predefined exercises and no typical progressions. In a pedagogy of success built on individual capacities, during each session and for each person, it is imperative to specifically program exercises that solicit individual motor resources. This is possible on the one hand thanks to a small number of practitioners and on the basis of an initial low level of motor skills, and on the other hand thanks to precise programming following the rigorous evaluation of individual functional abilities.

Understanding the influence of the initial level of functional reserves in the restoration of motor skills is a problem to be explored. The World Health Organization recently proposed an innovative model of healthy aging that articulates functional ability, the environment and the interactions between the two. Cesari et al. (2018) considered that “functional ability is determined by the intrinsic capacity (physical and mental) and in reference of the International Classification of Functioning, Disability and Health (ICF) framework, five domains (i.e, locomotion, vitality, cognition, psychological, sensory) are pivotal for capturing the individual’s intrinsic ability and therefore also reserves”. This preoccupation with individual functional reserves serves the prevention of frailty, and various works seek the validation of tools for screening for clinical signs of significant decrease in functional ability [32, 35, 37, 63]. The identification of reserves could also serve the rehabilitation of people with loss of autonomy and help in the definition of physical reconditioning planning models, taking into account the initial level and also the ability to progress.

This study included a number of limitations. In an interventional study, it would have been necessary to have a control group and to carry out a randomized-controlled study. The sample size (338 people), requiring a bootstrapping method to improve the power of the analysis, is a limitation. The specification in sub-profiles, qualified as low and middle level of balance, represented respectively only 14 and 12% of the total population. Larger recruitment with low-level equilibrium profiles should be a perspective in a future study. Another limitation is the relationship between assessment tasks and postural and balance reconditioning exercises reported during the 12-week training program. Although assessment tasks were not included in the training sessions, there is a close relationship between the assessment conditions and the motor skills mobilized during the adapted physical activity sessions [64]. This practice encouraged the learning and control of different types of movement, such as postural regulations in people with the weakest abilities at the beginning of the program. They practiced some adapted exercises centered on the perturbation of basic balance before engaging in more complex and intensive situations. They included locomotion and coordination tasks that solicited more intensive kinesthetic and muscular-articular dimensions. The use of a randomized-controlled study would allow the creation of several groups and yield a more precise work (i) on the notions of training individualization with regard to the functional capacities and (ii) on the management of the concepts of dose-response [65, 66]. The characteristics of the three-month PBM program with two 90-min weekly sessions, in groups of approximately ten people, may represent a limitation and warrant further study with other characteristics such as duration, frequency and driving practice contents.

## Conclusions

This study confirms the effect of a three-month physical conditioning PBM-HE program, with kinesthetic, postural and muscular-articular solicitations and information on health education to optimize the balance capacities of sedentary older adults who are at risk of falling. The second part of this study hypothesized that people with lower balance capacities would have a significantly limited progression. This assumption is partially invalid with the observation of a significant increase in balance capacities in older adults with a low level of balance, exclusively on basic motor behaviors and basic balance capacities. Future studies are still needed to identify specific training planning models that serve to optimize the functional reserves of the weakest people.
